# Supplementary material for: Latent-TGF-β has a domain swapped architecture
Source: Nat Commun. 2025 Nov 25;16:10469. doi: 10.1038/s41467-025-65465-w (PMC12647607; doi:10.1038/s41467-025-65465-w)
Supplement: Supplementary file 1 — Supplementary Information [file 41467_2025_65465_MOESM1_ESM.pdf]

Supplementary Information for:

Latent-TGF-β has a domain swapped architecture

Mingliang Jin[1,4], Robert Seed[2,4], Tiffany Shing[2], Li Wang[2], Junrui Li[3], Yifan Cheng[1,3*], Stephen L. Nishimura[2*]

[1]Department of Biochemistry and Biophysics, University of California San Francisco, San Francisco, CA, USA
[2]Department of Pathology, University of California San Francisco, San Francisco, CA, USA
[3]Howard Hughes Medical Institute, University of California San Francisco, San Francisco, CA, USA

[4] These authors contributed equally
*Correspondence: Stephen.Nishimura@ucsf.edu and Yifan.Cheng@ucsf.edu

Supplementary information includes:
Supplementary Fig. 1–3
Uncropped and unprocessed versions of SDS-PAGE gels

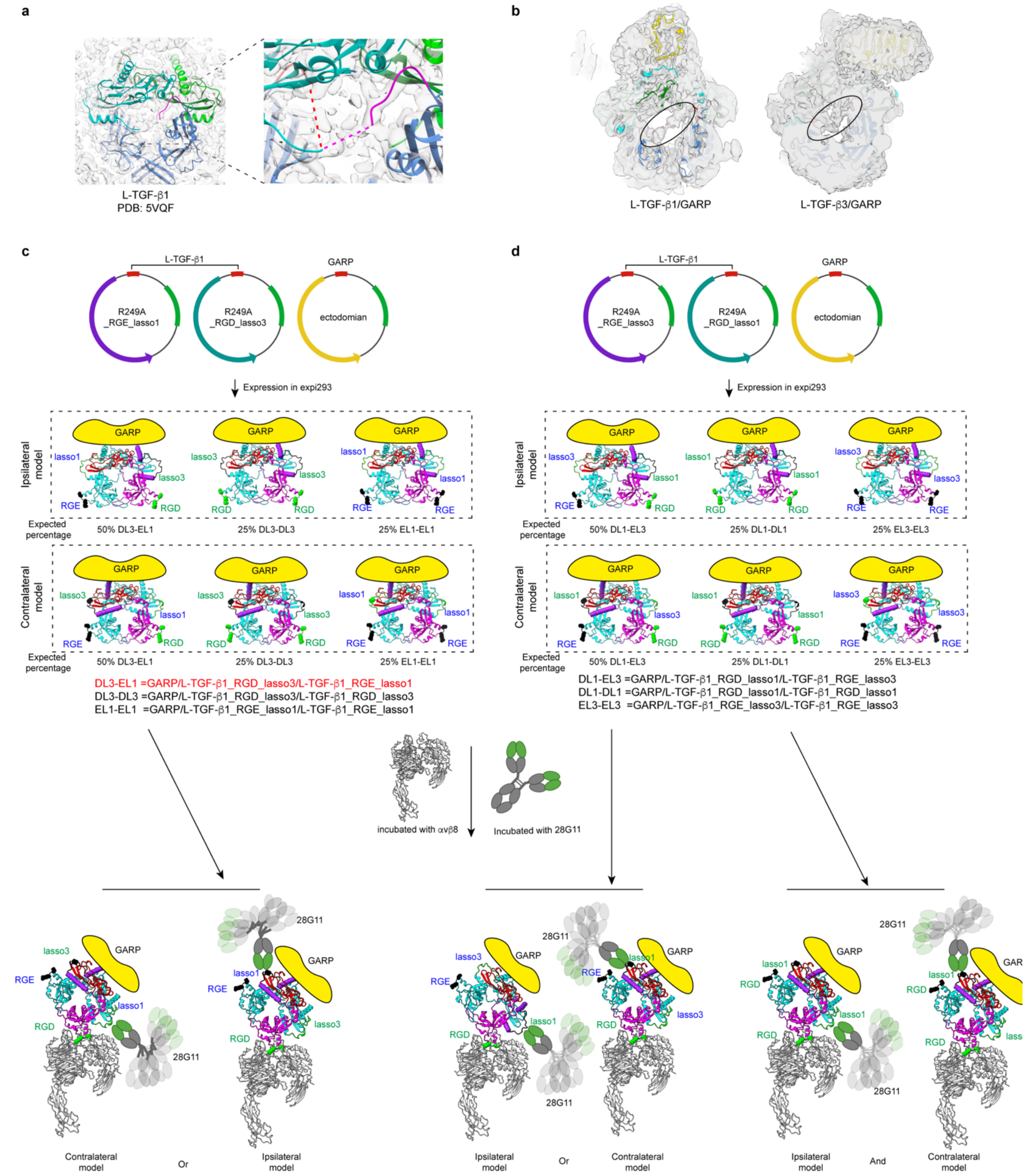

Supplementary Fig. 1 | Experimental design to determine the domain architecture of L-TGF-β1/GARP **a,** Calculated electron density map of L-TGF-β1 crystal structure (PDB: 5VQF) fitted with the atomic model. The enlarged view shows the density of the connector region is incompletely resolved, with the red dashed line linking the chains in an ipsilateral configuration. Magenta dashed line illustrates a possible alternative domain linkage for a contralateral

configuration. **b**, Cryo-EM density map of L-TGF-β1/GARP (left) and L-TGF-β3/GARP (right) without sharpening[5] are shown at low contour level, showing a weak density (circled in black) suggesting the domain linkage for a contralateral configuration. **c,** Rationale for mutant constructs used in this study. Plasmid construct cartoons (red and green indicate plasmid backbone) of L-TGF-β1_R249A_RGE_lasso1 (left, purple), L-TGF-β1_R249A_RGD_lasso3 (middle, teal) and GARP (right, yellow). Anticipated expression products and their proportions from the 1:1:1 transfection of Expi293 cells. Nomenclature of the expected products are shown (DL3-EL1=GARP/L-TGF-β1_RGD_lasso3/L-TGF-β1_RGE_lasso1; DL3-DL3=GARP/L-TGF-β1_RGD_lasso3/L-TGF-β1_RGD_lasso3; EL1-EL1 = GARP/L-TGF-β1_RGE_lasso1/L-TGF-β1_RGE_lasso1). DL3-EL1 is the desired complex and is highlighted in red. Ribbon diagrams of L-TGF-β1 in ipsilateral (upper) or contralateral (bottom) architectures are predicted by AlphaFold2, with wild type (wt) and mutant (mt) composition indicated below. The experimental design allows the unambiguous assignment of ipsilateral or contralateral prodomain monomers based on the relative positions of the 28G11 antibody and integrin αvβ8 in the L-TGF-β1/GARP DL3-ELI complex (below left). **d,** The same experimental design does not work with the RGE/lasso3 mutant, illustrating why this construct was not used in this study. Shown are the plasmid constructs of L-TGF-β1_R249A_RGE_lasso3 (left, purple) L-TGF-β1_R249A_RGD_lasso1 (middle, teal) and GARP (right, yellow) and the anticipated expression products and their proportions from the 1:1:1 transfection of Expi293 cells. Nomenclature of the expected products are shown (DL1-EL3 = GARP/L-TGF-β1_RGD_lasso1/L-TGF-β1_RGE_lasso3; DL1-DL1 = GARP/L-TGF-β1_RGD_lasso1/L-TGF-β1_RGD_lasso1; EL3-EL3 = GARP/L-TGF-β1_RGE_lasso3/L-TGF-β1_RGE_lasso3). The presence of WT L-TGF-β (DL1-DL1) in the expression products will allow αvβ8 and 28G11 to bind to each monomer of L-TGF-β and thus cannot discriminate between ipsilateral and contralateral domain architectures (below right). Ribbon diagrams of L-TGF-β1 in ipsilateral (upper) or contralateral (bottom) architectures are predicted by AlphaFold2, with wild type (wt) and mutant (mt) composition indicated below.

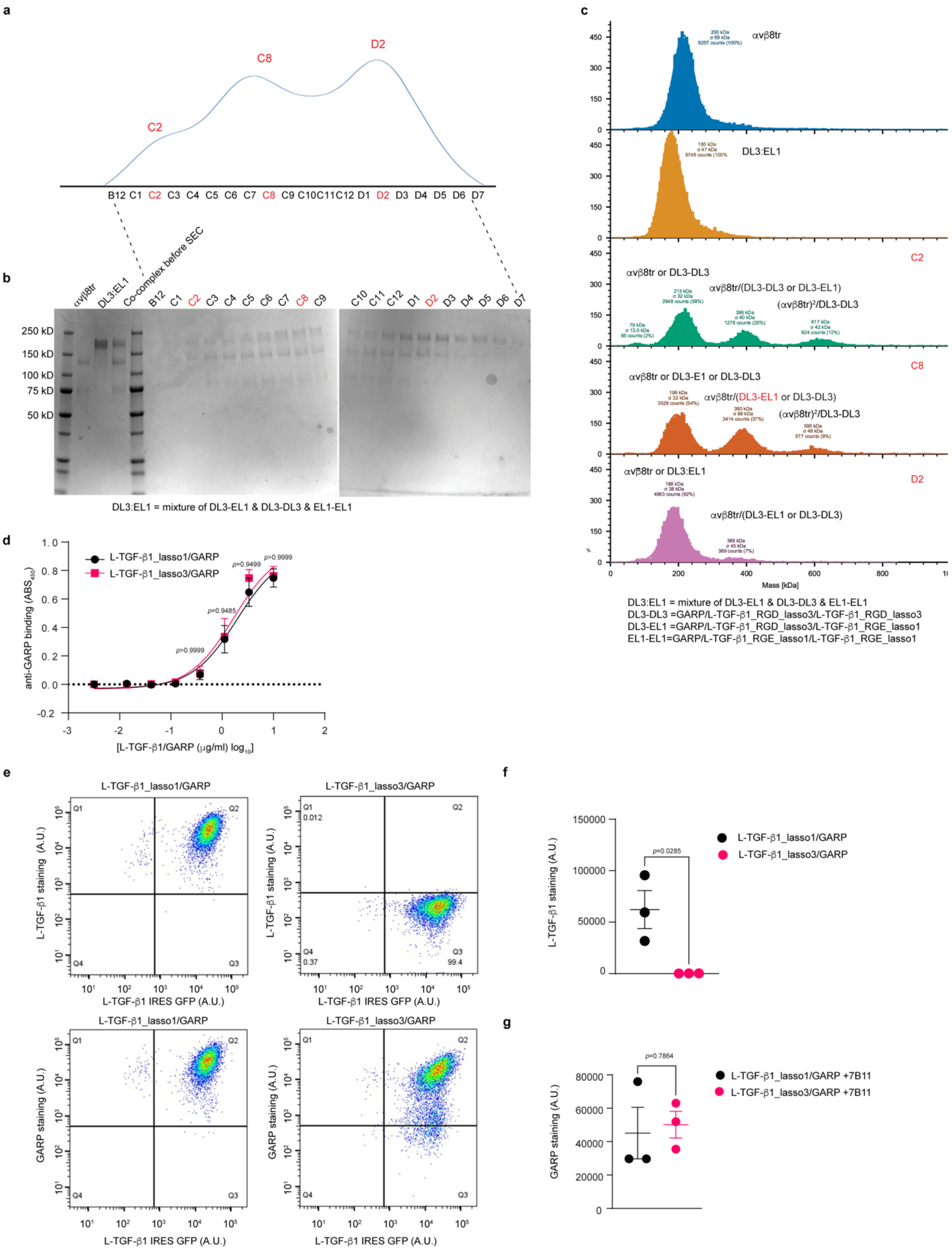

Supplementary Fig. 2 | Reconstitution of the mutant αvβ8/L-TGF-β1/GARP complex **a,** Size exclusion chromatography of the mutant αvβ8/L-TGF-β1/GARP complex. Protein fractions were

collected automatically, and absorption monitored at 280 nm. **b,** The indicated fractions were visualized by SDS-PAGE and Coomassie blue staining. Proteins and chosen fractions are indicated on the top. Note that only gel filtration peak fractions are shown. DL3:EL1 refers to a mixture of mutant forms DL3-EL1; DL3-DL3; EL1-EL1). The source data are shown below. **c,** Mass photometry analysis of mutant L-TGF-β1/GARP mixed with ectodomain of αvβ8. Peaks correspond to one L-TGF-β1/GARP bound with two αvβ8 integrins at ~600 kDa (fraction C2), one L-TGF-β1/GARP bound with one αvβ8 at ~390 kDa (fraction C8), and L-TGF-β1/GARP ~185 kDa or αvβ8 alone at ~200 kDa (fraction D2). Nomenclature of the expected mutant species are shown (DL3-EL1 = GARP/L-TGF-β1_RGD_lasso3/L-TGF-β1_RGE_lasso1; DL3-DL3 = GARP/L-TGF-β1_RGD_lasso3/L-TGF-β1_RGD_lasso3; EL1-EL1 = GARP/L-TGF-β1_RGE_lasso1/L-TGF-β1_RGE_lasso1). DL3-EL1 is the desired complex and is highlighted in red. Sample tracings from each samples/complexes are colored arbitrarily in different colors. **d,** ELISA using anti-GARP (clone 7B11) confirms that recombinant preparations of L-TGF-β1/GARP and L-TGF-β1 (lasso3)/GARP were immobilized at equal concentrations. Data represents mean ± SEM (N=4 biological replicates), *P*-values presented were generated via 2 way ANOVA with repeated measures and Šídák's multiple comparisons test. **e,** Representative flow cytometry scatter plots demonstrating the percentage of cell surface L-TGF-β1_lasso1 (lower, left), L-TGF-β1_lasso3 (lower, right), as measured by anti-GARP (7B11) and eGFP dual positive staining (upper right quadrants), and the related binding properties of 28G11 (upper row). Data represents a single example from 3 independent experiments. **f,g,** 28G11 and 7B11 binding, respectively. Data is presented as mean ±SEM of mean fluorescence intensity in arbitrary units (*A.U.*). N=3 biological replicates, *p<0.05 by students T-test. Source data is available in the source data table.

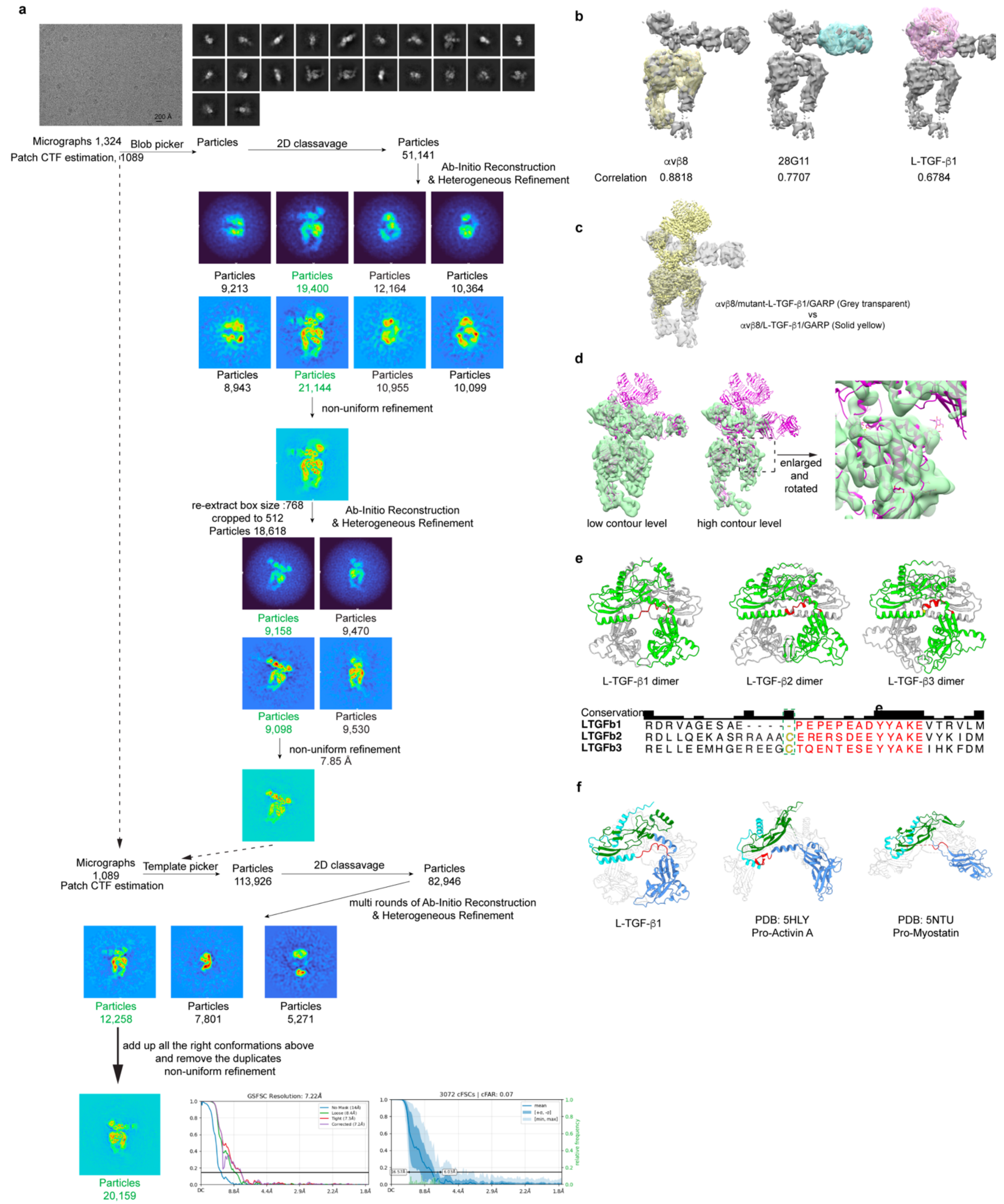

Supplementary Fig. 3 | Single particle cryo-EM of mutant L-TGF-β1/GARP/28G11 complex

a, Single particle cryo-EM data processing pipeline of αvβ8/L-TGF-β1/GARP/28G11. **b**, Atomic model and simulated map of αvβ8, L-TGF-β1, and a Fab was fit into the corresponding density of

the map. Cross-correlation between the simulated and the corresponding density of the experimental map are shown below. **c**, The cryo-EM map of αvβ8/L-TGF-β1/GARP[5] was overlayed with the experimental map. **d**, The final map of αvβ8/mutant L-TGF-β1/GARP/28G11 was low pass filtered to 8 Å with an atomic model docked. **e**, Alphafold2 predicted structure of L-TGF-β1, 2 and 3 in contralateral form, linkers are indicated in red. Below, are sequence alignments and conservation of the a2-helix, and linker for each TGF-β isoform in red type with the cysteines in TGF-β2 and -β3 in green dashed box. **f**, Alphafold predicted monomer structures of L-TGF-β1 (left), compared to monomers from crystal structures of Pro-Activin A (PDB: 5HLY, middle), and Pro-Myostatin (PDB: 5NTU, right) highlighting the domain-swapped architecture with the connectors in red.

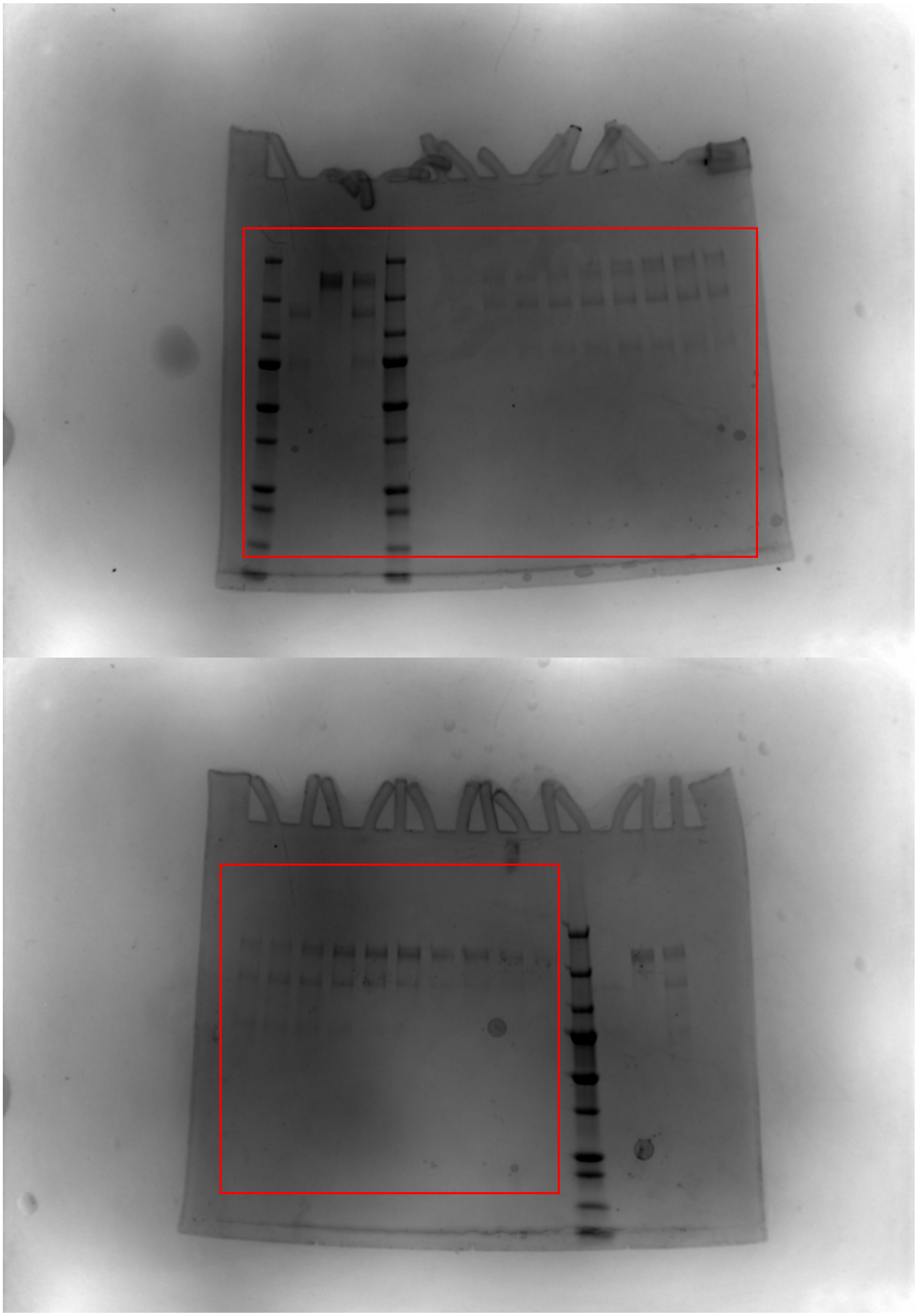

Source Data for Supplementary Fig. 2b | Uncropped and unprocessed versions of SDS-PAGE gels relevant to **Supplementary Fig. 2b**. The areas shown are indicated by red rectangles.
