# Supplementary material for: Latent-TGF-β has a domain swapped architecture
Source: Nat Commun. 2025 Nov 25;16:10469. doi: 10.1038/s41467-025-65465-w (PMC12647607; doi:10.1038/s41467-025-65465-w)
Supplement: Supplementary file 2 — Reporting Summary [file 41467_2025_65465_MOESM2_ESM.pdf]

nature portfolio

Corresponding author(s): Yifan Cheng and Stephen Nishimura

Last updated by author(s): Sep 25, 2025

Reporting Summary

Nature Portfolio wishes to improve the reproducibility of the work that we publish. This form provides structure for consistency and transparency in reporting. For further information on Nature Portfolio policies, see our Editorial Policies and the Editorial Policy Checklist.

Statistics

For all statistical analyses, confirm that the following items are present in the figure legend, table legend, main text, or Methods section.

n/a	Confirmed	
☐	☒	The exact sample size (n) for each experimental group/condition, given as a discrete number and unit of measurement
☐	☒	A statement on whether measurements were taken from distinct samples or whether the same sample was measured repeatedly
☒	☐	The statistical test(s) used AND whether they are one- or two-sided<br>*Only common tests should be described solely by name; describe more complex techniques in the Methods section.*
☒	☐	A description of all covariates tested
☒	☐	A description of any assumptions or corrections, such as tests of normality and adjustment for multiple comparisons
☒	☐	A full description of the statistical parameters including central tendency (e.g. means) or other basic estimates (e.g. regression coefficient) AND variation (e.g. standard deviation) or associated estimates of uncertainty (e.g. confidence intervals)
☒	☐	For null hypothesis testing, the test statistic (e.g. F, t, r) with confidence intervals, effect sizes, degrees of freedom and P value noted<br>*Give P values as exact values whenever suitable.*
☒	☐	For Bayesian analysis, information on the choice of priors and Markov chain Monte Carlo settings
☒	☐	For hierarchical and complex designs, identification of the appropriate level for tests and full reporting of outcomes
☒	☐	Estimates of effect sizes (e.g. Cohen's d, Pearson's r), indicating how they were calculated

Our web collection on statistics for biologists contains articles on many of the points above.

Software and code

Policy information about availability of computer code

Data collection	Serial EM was used for cryo-EM data collection.
Data analysis	cryoSPARC v4, MOTIONCOR2, Chimera v1.16.0, ChimeraX v1.4, ALPHAFOLD, GraphPad Prism, Colab webserver

For manuscripts utilizing custom algorithms or software that are central to the research but not yet described in published literature, software must be made available to editors and reviewers. We strongly encourage code deposition in a community repository (e.g. GitHub). See the Nature Portfolio guidelines for submitting code & software for further information.

Data

Policy information about availability of data

All manuscripts must include a data availability statement. This statement should provide the following information, where applicable:

- Accession codes, unique identifiers, or web links for publicly available datasets
- A description of any restrictions on data availability
- For clinical datasets or third party data, please ensure that the statement adheres to our policy

Cryo-EM map of mutant avb8/L-TGF-b/GARP/28G11 is deposited to EMDB with accession number EMDB-47130.

Research involving human participants, their data, or biological material

Policy information about studies with human participants or human data. See also policy information about sex, gender (identity/presentation), and sexual orientation and race, ethnicity and racism.

Reporting on sex and gender	N/A
Reporting on race, ethnicity, or other socially relevant groupings	N/A
Population characteristics	N/A
Recruitment	N/A
Ethics oversight	N/A

Note that full information on the approval of the study protocol must also be provided in the manuscript.

Field-specific reporting

Please select the one below that is the best fit for your research. If you are not sure, read the appropriate sections before making your selection.

[x] Life sciences [ ] Behavioural & social sciences [ ] Ecological, evolutionary & environmental sciences

For a reference copy of the document with all sections, see nature.com/documents/nr-reporting-summary-flat.pdf

Life sciences study design

All studies must disclose on these points even when the disclosure is negative.

Sample size	Sample sizes for cell based assays were chosen based on power to detect differences of approximately 50% between groups. This generally required a minimum of 3 biologic replicate.
Data exclusions	No data was excluded for cell-based assays
Replication	All reported data had a minimum of 3 biologic replicates
Randomization	Randomization was not used.
Blinding	Samples were not blinded.

Reporting for specific materials, systems and methods

We require information from authors about some types of materials, experimental systems and methods used in many studies. Here, indicate whether each material, system or method listed is relevant to your study. If you are not sure if a list item applies to your research, read the appropriate section before selecting a response.

Materials & experimental systems

n/a	Involved in the study	
[ ]	[x]	Antibodies
[ ]	[x]	Eukaryotic cell lines
[x]	[ ]	Palaeontology and archaeology
[x]	[ ]	Animals and other organisms
[x]	[ ]	Clinical data
[x]	[ ]	Dual use research of concern
[x]	[ ]	Plants

Methods

n/a	Involved in the study	
[x]	[ ]	ChIP-seq
[x]	[ ]	Flow cytometry
[x]	[ ]	MRI-based neuroimaging

Antibodies

Antibodies used	anti-LAP (TW-28G11, BioLegend), anti-GARP (7B11, BioLegend), and anti-mouse-APC conjugated secondary antibody (Biolegend #405308) are commercial. The Nishimura Laboratory created anti-av (8B8) .
Validation	These commercial antibodies are validated by the companies. anti-av (8B8) is used to purify avb8.

Eukaryotic cell lines

Policy information about cell lines and Sex and Gender in Research

Cell line source(s)	expi293 (Thermofisher), expiCHO (Thermofisher), Transformed Mink Lung Epithelial Cells (Gift of John Munger, NYU, NYC, NY)
Authentication	None of the cells were authenticated
Mycoplasma contamination	No contamination is detected in all the cell lines.
Commonly misidentified lines (See ICLAC register)	None of the lines used are on the ICLAC register.

Plants

Seed stocks	N/A
Novel plant genotypes	N/A
Authentication	N/A
